# Case Report: POEMS syndrome secondary to multiple solitary plasmacytomas complicated by hypertriglyceridemia

**DOI:** 10.3389/fmed.2025.1725102

**Published:** 2026-01-05

**Authors:** Wenchao Chen, Fang Xue, Na Wang, Zilong Zhang, Cunshui Xue, Xia Lian

**Affiliations:** 1 Department of Neurology, The Second Hospital of Shanxi Medical University, Taiyuan, China; 2 Department of Rehabilitation Medicine, The First Hospital of Shanxi Medical University, Taiyuan, China; 3 Department of Neurology, Shanxi Medical University, Taiyuan, China

**Keywords:** case report, hypertriglyceridemia, neuropathy, plasmacytoma, POEMS syndrome

## Abstract

**Introduction:**

Polyneuropathy, organomegaly, endocrinopathy, M protein, skin changes syndrome, encompassing polyneuropathy, organomegaly, endocrinopathy, M protein (Monoclonal Immunoglobulin), and skin changes, is a rare systemic disease often marked by diverse endocrine manifestations. Diagnosis typically relies on the detection of monoclonal protein; therefore, cases lacking measurable M-protein may be easily overlooked or misdiagnosed.

**Case report:**

We describe a diagnostically challenging case involving a patient in their early fifties who presented with progressive limb numbness and weakness, skin hyperpigmentation, lower-extremity edema, hypothyroidism, and marked hypertriglyceridemia. Despite this multisystem involvement, repeated serum and bone marrow studies failed to demonstrate monoclonal protein. Subsequent imaging and pathological evaluation of the sacroiliac region revealed multiple solitary plasmacytomas with lambda light-chain restriction, which established the diagnosis of POEMS syndrome in the absence of detectable M-protein. The patient experienced notable clinical improvement following bortezomib-based chemotherapy.

**Conclusion:**

This case illustrates that POEMS syndrome should be considered even when serum and bone marrow studies do not show monoclonal protein, particularly in the presence of severe hypertriglyceridemia and multisystem injury. Awareness of such atypical presentations is essential to prevent diagnostic delay and to ensure timely initiation of appropriate therapy.

## Introduction

Polyneuropathy, organomegaly, endocrinopathy, M protein, skin changes (POEMS) syndrome represents a rare systemic disorders (1). In 1980, Bardwic introduced the acronym “POEMS” to encapsulate the clinical manifestations of this syndrome (2). While the acronym POEMS effectively encapsulates the clinical manifestations of this disorder, certain crucial clinical features remain unrepresented. Notably absent from the acronym are significant aspects such as sclerotic bone lesions, Castleman disease, papilledema, pleural effusion, edema, ascites, and thrombocytosis. The diagnosis of POEMS syndrome lacks a singular diagnostic assay. Instead, a definitive determination relies upon the meticulous correlation of discernibly distinct symptoms and clinical indicators (3).

In this case report, we describe an atypical presentation of POEMS syndrome characterized by pronounced hypertriglyceridemia and the presence of multiple solitary plasmacytomas, both of which contributed to the diagnostic complexity. Although hypertriglyceridemia is not part of the established diagnostic criteria for POEMS, it may indicate underlying endocrine dysfunction–such as hypothyroidism–or other metabolic disturbances associated with the syndrome. By presenting this case, we aim to draw attention to these uncommon yet clinically meaningful features and to underscore the importance of recognizing POEMS presentations that fall outside the traditional diagnostic criteria.

This case report was approved by the Ethics Committee of the Second Hospital of Shanxi Medical University (Approval No.: 2022YX143), and the patient provided written informed consent for all treatments performed and to publish this case report. The reporting of this study conforms to CARE guidelines (4).

### Case description

**FIGURE 1:**
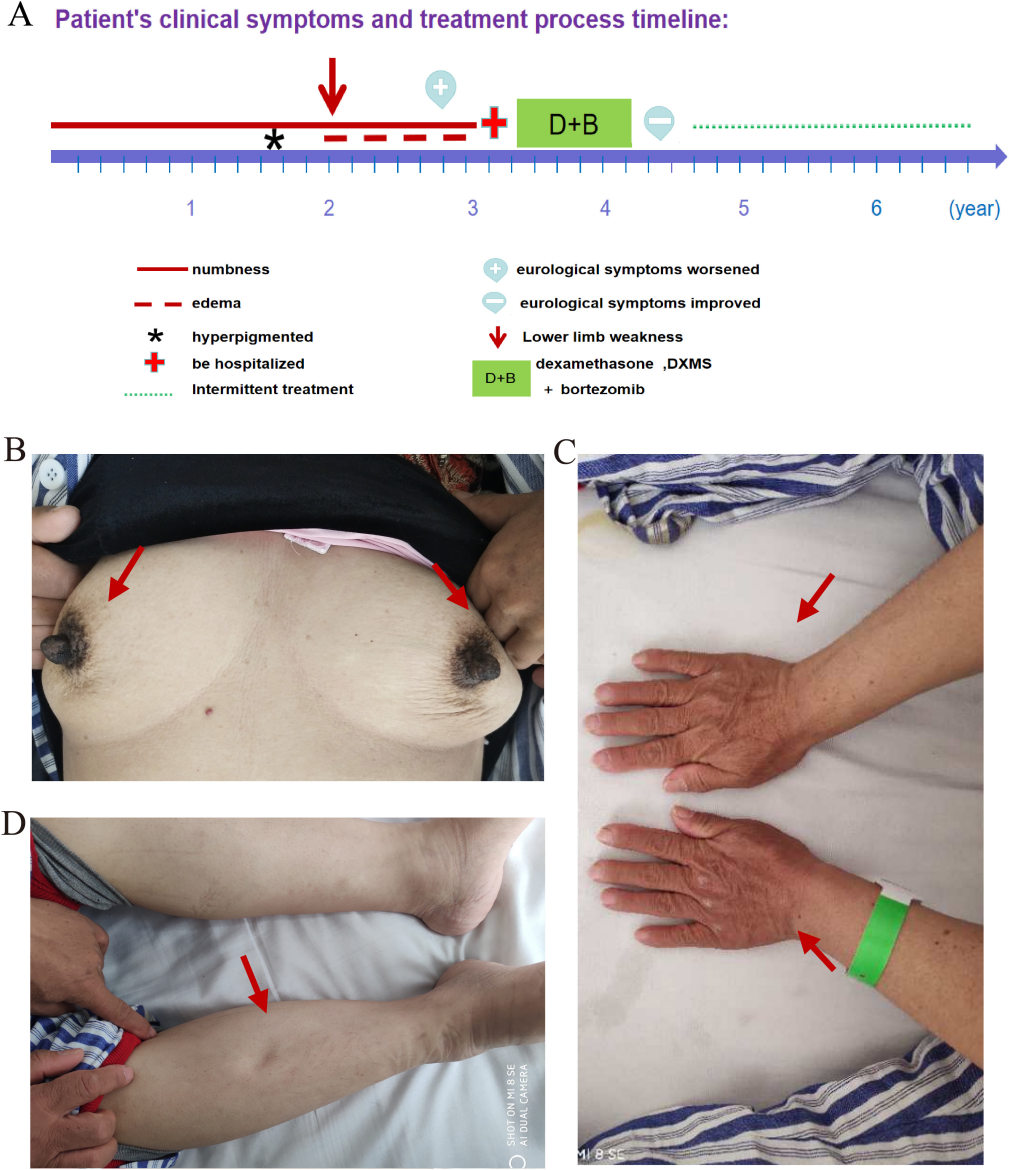
**(A)** The timeline of the patient’s symptom onset and subsequent diagnostic and therapeutic processes. **(B–D)** The patient’s skin changes. **(B)** Close-up view showing increased pigmentation in the patient’s areola (red arrow), indicating a marked darkening of the area compared to surrounding skin. **(C)** Detailed view of the patient’s hands, with pronounced hyperpigmentation over the knuckles and dorsal surface (red arrow). **(D)** Lower limb examination revealing pitting edema (red arrow), demonstrating indentation upon pressure application and suggesting fluid retention in the affected area.

Our hospital admitted an early 50s female patient with a relatively complex medical history (Figure 1A). The main symptoms included intermittent numbness in the hands for over 3 years and numbness and weakness in both lower limbs for 1 year.

Three years ago, the patient experienced episodes of hand numbness lasting approximately 30 min. Methylcobalamin tablets was administered at the local hospital to support nerve health, which alleviated the symptoms. A year ago, she developed bilateral lower limb edema accompanied by numbness, leg weakness, unstable gait, a sensation of walking on cotton, and increased cold sensitivity. The local hospital performed cervical CT and MRI scans to exclude cervical spondylopathy and considered the diagnosis to be polymyositis, while not ruling out the possibility of a malignant tumor. Over time, her condition deteriorated, with worsening bilateral lower limb weakness, rendering her unable to walk independently.

Upon presentation at our hospital, the patient exhibited clear consciousness and fluent speech. She is 1.58 m tall and weighs approximately 48 kg (BMI 19.2 kg/m^2^), presenting a markedly thin appearance. Examination reveals prominent hyperpigmentation on the face, hands, and upper limbs, with notably darker skin on the hands and darkened areolas (Figures 1B, C). Upper limb muscle strength was 5/5 on the MRC scale, with normal muscle tone. Lower limb muscle strength was 3/5, with preserved tendon reflexes and negative bilateral Babinski signs. Superficial and vibrational sensations below the knees were diminished. Enlarged lymph nodes were observed in the neck, supraclavicular, and axillary regions. Edema with pitting was observed in the lower extremities (Figure 1D). No abnormalities were detected in the cardiovascular, respiratory, or abdominal systems.

Notably, prior to symptom onset, she maintained good health, a stable lifestyle, and had no family history of similar symptoms, familial hypertriglyceridemia, or relevant genetic disorders.

### Imaging and functional examinations

**FIGURE 2:**
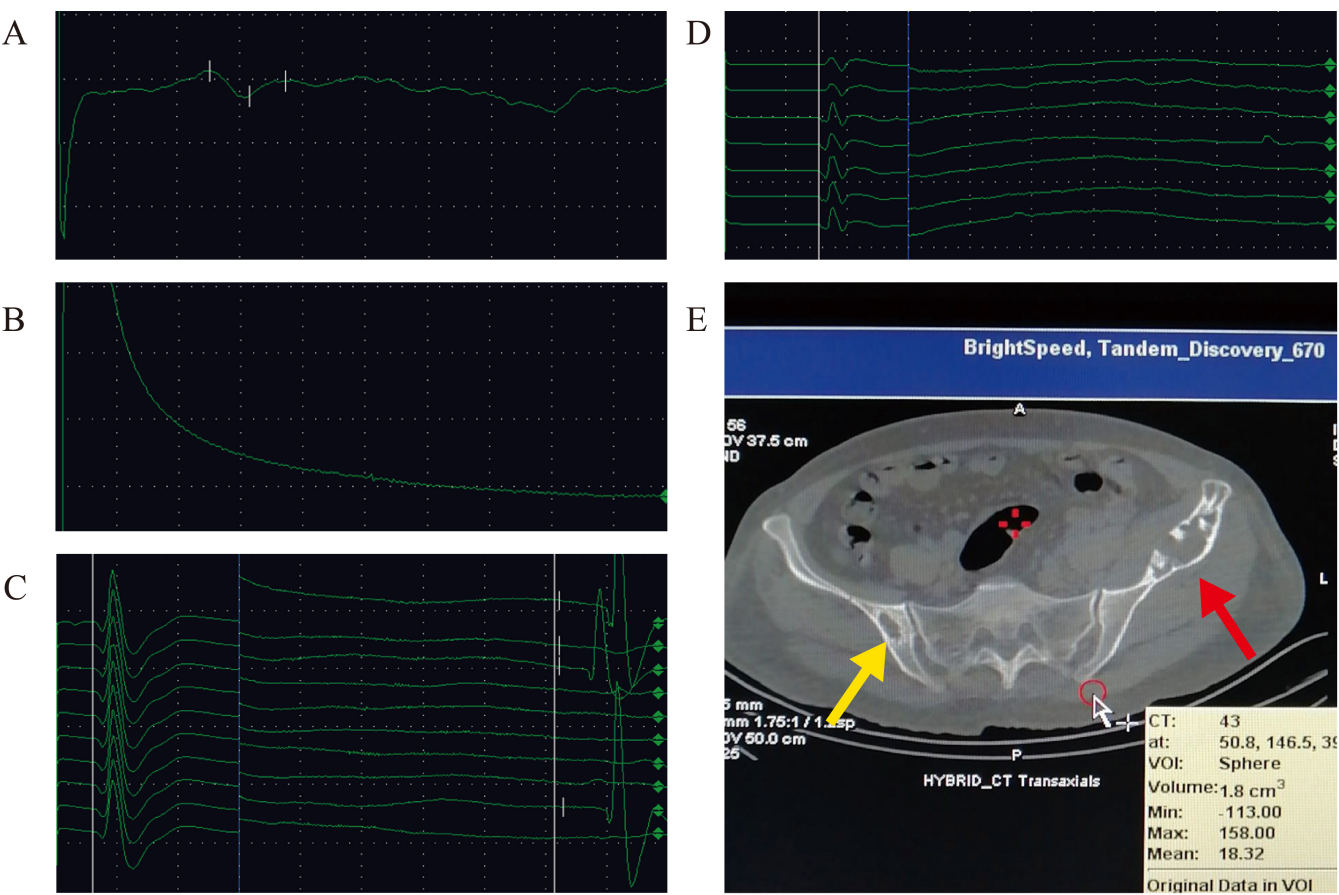
Electromyography examination and sacroiliac joint CT scan. **(A)** The sensory conduction velocity of the left ulnar nerve exhibited a marked reduction. **(B)** The sensory conduction velocity testing of the right peroneal nerve failed to evoke positive waveforms. The F-waves of the left ulnar nerve **(C)** and left median nerve **(D)** were not elicitable **(E)**. CT scan of the sacroiliac joint (refer to annotations in the lower right corner): limited linear high-density shadows (yellow arrow) are observed on the right sacroiliac joint iliac bone surface, with irregular low-density shadows suggesting bone destruction and a “doughnut-shaped” distribution of contrast agent, accompanied by local sclerosis. On the left iliac wing, expansive osteolytic lesions are evident (red arrow), with adjacent thinning of the cortical bone. Spotty calcifications are noted within, and there is no apparent soft tissue mass in the surrounding area. The tissue biopsy site is also indicated by the red arrow, which points to the soft tissue mass on the left side.

Abdominal ultrasound revealed hepatic steatosis, with no apparent enlargement of abdominal lymph nodes. Cardiac color Doppler ultrasound showed a small amount of pericardial effusion. Superficial lymph node ultrasonography showed bilateral neck, left supraclavicular fossa, and bilateral axillary lymphadenopathy. The electromyography results showed a significant reduction in the sensory conduction velocity of the left ulnar nerve, while the sensory conduction velocity testing of the right peroneal nerve failed to evoke positive waveforms; F-waves of the left ulnar nerve and left median nerve were not elicitable (Figures 2A–D). Bone marrow aspiration and biopsy were performed at the posterior iliac crest, with results revealing no pathological abnormalities.

Bone scan revealed that the left iliac wing and right iliac near the sacroiliac joint showed abnormal active bone metabolism. CT scan of the sacroiliac joint revealed bilateral iliac wing bone destruction and soft tissue mass (Figure 2E), along with bilateral sacroiliac joints degenerative changes. Needle biopsy of the soft tissue obtained from the left sacroiliac joint revealed diffuse proliferation and infiltration of plasma-like cells.

### Laboratory tests

Vascular endothelial growth factor (VEGF): 800 pg/mL, TNF-α: 3.94 pg/mL and triglycerides level: 9.06 mmol/L. No M protein was detected in the serum and urine, and the results do not support a diagnosis of monoclonal plasma cell disorder. We conducted further communication with the patient and proceeded with immunofixation electrophoresis testing. The results showed a serum κ (kappa) light chain level of 6.57 g/L (within the normal range) and a λ (lambda) light chain level of 12.90 g/L (elevated). In the urine, the κ (kappa) light chain was 10.80 mg/L (elevated), and the λ (lambda) light chain was 6.44 mg/L (elevated). The key laboratory test results and reference ranges are provided in Table 1.

**TABLE 1:** Summary of laboratory test data.

Category	Parameter	Unit	Reference range	At admission	After treatment
Hematology & biochemistry	WBC	×10^9^/L	4.0–11.0	10.60	7.68
	Hb	g/L	120–150	175	164
	PLT	×10^9^/L	125–350	484	327
	TG	mmol/L	0.56–1.7	**9.06**	**4.54**
	tHCY	μmol/L	5–15	**18.60**	13.75
	HbA1c	%	4–6	**6.90**	**6.50**
Urine & serum light chains	Light chain κ (urine)	mg/L	0–7.099	**10.80**	**9.75**
	Light chain λ (urine)	mg/L	0–3.899	**6.44**	**6.18**
	Light chain κ	g/L	6.29–13.5	6.57	5.33
	Light chain λ	g/L	3.13–7.23	**12.90**	**9.45**
CSF parameters	IgG (CSF)	mg/L	0–34	**137.00**	**84**
	IgA (CSF)	mg/L	0–8	**9.37**	8.54
	Protein (CSF)	g/L	0.15–0.45	**0.60**	**0.47**
	WBC (CSF)	×106/L	0–5	4	3.8
Associated bioactive molecules	IgG	g/L	7–16	**16.60**	14.35
	TNF-α	pg/mL	0.10–2.31	**3.94**	**3.52**
	VEGF	pg/mL	0–142.2	**800**	**635**
Endocrine parameters	E2	pmol/L	79.4–146.8	**305.00**	**274**
	PRL	mIU/L	102–496	**683.80**	**592.40**
	FT3	mol/L	3.8–6.0	4.15	5.31
	FT4	mol/L	7.9–14.4	9.12	12.46
	TSH	mIU/L	0.35–4.94	**5.77**	**5.38**

WBC, white blood cell count; Hb, hemoglobin; PLT, platelet count; TG, triglycerides; tHCY, total homocysteine; HbA1c, glycated hemoglobin; κ/λ, Kappa/Lambda light chains; IgG/IgA, immunoglobulin G/A; Protein (CSF), protein in cerebrospinal fluid; TNF-α, Tumor Necrosis Factor alpha; VEGF, vascular endothelial growth factor; E2, estradiol; PRL, prolactin; FT3, free triiodothyronine; FT4, free thyroxine; TSH, thyroid stimulating hormone. Values outside the reference range are highlighted in bold.

**FIGURE 3:**
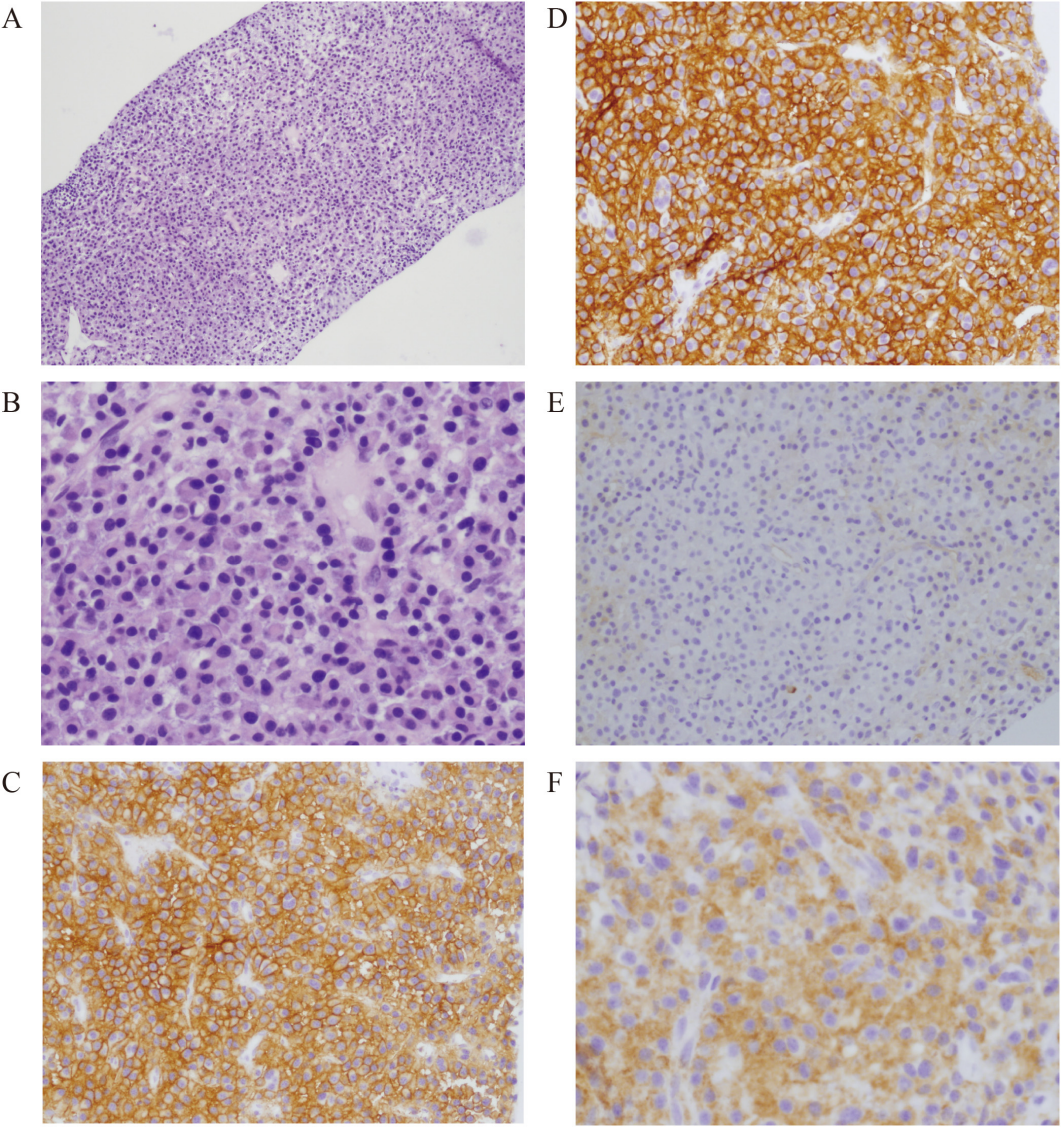
Soft tissue biopsy of the sacroiliac joint shows lambda type plasmacytoma. **(A)** HE, ×100; **(B)** HE, ×400: HE staining shows diffuse proliferation and infiltration of plasma-like cells, rich cytoplasm, red staining, and no nucleoli. **(C)** CD38, ×200; **(D)** CD138, ×200; **(E)** Kappa, ×200; **(F)** Lambda, ×200: immunohistochemistry examination shows CD3 (–), CD20 (–), PAX-5 (–), Ki67 (5% +), CD38 (+), CD138 (+), MUM1 (+), Kappa (–), Lambda (+), Cyclin D1 (–), consistent with the lambda type plasma cell tumor.

Hematoxylin and Eosin (HE) staining exhibited widespread proliferation and infiltration of plasma-like cells with abundant cytoplasm, displaying a red hue and lacking nucleoli. The immunohistochemical result was consistent with lambda type plasmacytoma (Figures 3A–F).

### Diagnosis and treatment

The patient was finally diagnosed with POEMS syndrome with multiple solitary plasmacytomas (lambda type). She was administered chemotherapy with bortezomib combined with dexamethasone: bortezomib 2.2 mg, subcutaneously injected on days 1, 4, 8, and 11; dexamethasone 20 mg, intravenous infusion on days 1, 2, 4, 5, 8, and 9.

Following a single course of chemotherapy, significant improvements were observed in symptoms such as pigmentation, lower-limb weakness, walking capacity, and distal numbness in the lower limbs. Furthermore, there was a notable reduction in the triglyceride level, which decreased to 4.0 mmol/L.

The patient was followed up for a duration of 49 months. During this period, she underwent an additional two rounds of chemotherapy at a local medical facility. However, due to financial constraints, she elected to prematurely terminate the prescribed chemotherapy regimen. Upon the latest follow-up, her ambulatory capacity remained compromised, accompanied by diminished appetite and persistent skin pigmentation. No further laboratory assessments were conducted. The onset and diagnostic course of the patient’s condition are also depicted on the timeline graphic (Figure 1A).

## Discussion

Polyneuropathy, organomegaly, endocrinopathy, M protein, skin changes syndrome is a paraneoplastic disorder caused by an underlying plasma cell neoplasm (5). According to the current diagnostic criteria, the major features include polyneuropathy, a clonal plasma cell disorder (PCD), sclerotic bone lesions or mixed bone lesions, elevated vascular endothelial growth factor levels, and concurrent Castleman disease. Minor criteria consist of organomegaly, endocrinopathy, distinct skin changes, papilledema, extravascular volume overload, and thrombocytosis (6). Diagnosis requires meeting three main criteria, including polyneuropathy and clonal plasma cell disorder, and at least one secondary criterion. This patient fulfilled two essential criteria: multiple peripheral neuropathy and monoclonal plasma cell proliferative disorder. Additionally, the major criteria of sclerotic bone lesions and elevated VEGF levels were met, alongside several minor criteria including hepatomegaly, lymphadenopathy, edema, thyroid and gonadal involvement, and skin hyperpigmentation. Thus, a diagnosis of POEMS syndrome was confirmed.

Recent reports have described variant forms of POEMS syndrome that do not fully meet the established diagnostic criteria, creating significant diagnostic challenges. Some patients present with typical clinical features but lack detectable monoclonal protein in serum or urine, representing an “M-protein–negative” subtype (5, 7). Such observations suggest that strict dependence on M-protein as a mandatory criterion may result in missed diagnoses in a considerable proportion of patients (5). Rare cases without peripheral neuropathy–the other mandatory criterion–have also been documented (8). In patients with strong clinical suspicion who exhibit Castleman disease, markedly elevated VEGF levels, and multiple minor features, clinicians should consider the possibility of variant POEMS syndrome and initiate timely management to prevent diagnostic delay (7).

In this report, the patient was initially diagnosed with familial hypertriglyceridemia (FHTG) based on elevated triglyceride levels and normal cholesterol levels in blood tests. However, the patient had no family history of FHTG, and FHTG could not account for the symptoms of limb numbness and weakness or the abnormal electromyography findings. Therefore, we considered that the elevated triglyceride levels in the patient and the peripheral neuropathy may be disparate clinical manifestations stemming from a common etiology, bringing POEMS syndrome into our purview. Within the current research on the pathogenesis of POEMS syndrome, the pathogenic role of pro-inflammatory cytokines is widely acknowledged. Among these, the prolonged elevation of the pro-inflammatory cytokine TNF-α is recognized to lead to inflammatory demyelinating neuropathy and hypertriglyceridemia, aligning with the principal clinical symptoms observed in this patient. Consequently, we conducted further investigations related to POEMS syndrome to elucidate its association in this case. Subsequent tests showed elevated levels of κ and λ light chains in the urine. Bone scan and CT revealed iliac bone lesions, and further biopsy confirmed the presence of λ-type plasmacytoma. Taking into account the patient’s clinical presentation and examination outcomes, a diagnosis of POEMS syndrome was established.

Neuropathy is the main feature of POEMS. The quality and extent of neuropathy include peripheral, ascending, and symmetrical neuropathy, and simultaneously affect the sensory and motor functions (6). Guibert et al. emphasized that all POEMS patients demonstrate axonal loss characterized by demyelination features and an excessive expression of VEGF, which correlates with increased perineural vascularity, the latter being associated with loss of large nerve fibers (9). Keddie et al. recommends that all patients presenting with an inflammatory neuropathy be subjected to appropriate investigations to screen for the presence of a monoclonal plasma cell proliferative disorder (10). This patient’s initial symptoms of bilateral symmetrical numbness and weakness over a span of 3 years align with the characteristic neuropathy of POEMS. It is important to note that severe generalized edema may interfere with the accuracy of electrophysiological studies, temporarily obscuring objective evidence of peripheral neuropathy. In such cases, POEMS syndrome should not be excluded prematurely, and the diagnosis should be guided by other systemic manifestations (8).

In POEMS, “M” refers to monoclonal, a mandatory criterion. Keddie et al. suggested evaluating all inflammatory neuropathy patients for monoclonal plasma cell disorders. Evidence includes positive serum/urine immunofixation, abnormal light chain ratio, and confirming biopsy (10). In this case, serum and urine immunofixation electrophoresis results were positive. Further whole-body bone scan revealed bone lesions, and pathological examination indicated λ-type plasmacytoma. This underscores the significance of vigilance toward solitary plasmacytomas of bone (SPB) and local pathological biopsies in preventing misdiagnosis of POEMS. In patients with a high clinical suspicion of POEMS syndrome but negative M-protein results, other evidence of clonal plasma cells should be actively sought. Biopsies of sclerotic bone lesions or lymph nodes can sometimes reveal clonal plasma cells, supporting the diagnosis (5).

Skin alterations are prevalent in POEMS patients (3). While these changes do not impact prognosis, identifying these symptoms contributes to disease diagnosis. Hyperpigmentation and angiomas are the most frequent manifestations (11). Hyperpigmentation presents as excessive pigmentation on the face, hands, and areolae. Following treatment, symptoms of skin hyperpigmentation gradually alleviate. The skin presentation in this case patient aligns with the aforementioned characteristics.

A notable characteristic in this patient is elevated triglyceride levels, a relatively uncommon manifestation. Elevated Tumor Necrosis Factor-alpha (TNF-α) can activate hormone-sensitive lipase through phosphorylation, promoting peripheral fat mobilization and increasing TG levels. In this patient, triglyceride levels were markedly elevated upon admission, along with increased TNF-α levels. After treatment, triglyceride levels returned to near normal. Thus, swift recognition of severe hypertriglyceridemia and multi-system involvement is pivotal in clinical practice to discern potential POEMS syndrome. Consequently, in the realm of clinical practice, it is imperative to promptly diagnose individuals afflicted with severe hypertriglyceridemia and multi-system impairment to ascertain the potential presence of POEMS syndrome.

The pathogenesis of POEMS syndrome remains unclear. Based on research, mechanisms such as monoclonal plasma cell proliferation, 14q32 chromosomal translocations and 13q14 chromosomal deletions, Epstein-Barr virus and human herpesvirus 8 infections, along with excessive production of proinflammatory cytokines and VEGF, are thought to play significant roles in the onset and progression of POEMS syndrome (12).

The optimal treatment strategy for POEMS syndrome remains an ongoing area of investigation. For localized lesions, radiotherapy appears to hold promise as a viable treatment option (6). In cases of disseminated disease, systemic interventions are essential, encompassing melphalan with dexamethasone, bortezomib with cyclophosphamide or dexamethasone, as well as autologous stem cell transplantation (13). Moreover, four case studies encompassing a cohort of 12 patients, who received bortezomib either as a standalone therapeutic agent or in conjunction with dexamethasone or cyclophosphamide for the management of POEMS syndrome, have demonstrated variable alterations in hemogram parameters, VEGF levels, and mitigation of peripheral nerve symptoms (14). In the current case, the patient underwent treatment with a combination of bortezomib and dexamethasone, resulting in notable amelioration of symptoms. Recent clinical practice indicates an increasing diversification of treatment options. Lenalidomide combined with dexamethasone (the RD regimen), owing to its efficacy and tolerability, has become a first-line therapy in many centers (15). For relapsed or refractory cases, the next-generation proteasome inhibitor carfilzomib combined with dexamethasone (the KD regimen) has also demonstrated excellent efficacy and safety (16).

Thus, it is important to consider POEMS syndrome as a differential diagnosis in patients with severe hypertriglyceridemia and symptoms of multiple system involvement. For patients with suspected POEMS syndrome, if the M protein in the serum and bone marrow is negative, screening for skeletal lesions is essential. A whole-body bone scan may reveal infiltrative lesions, prompting localized biopsy for pathological confirmation to prevent misdiagnosis.

## Data Availability

The original contributions presented in this study are included in this article/supplementary material, further inquiries can be directed to the corresponding author.
